# Research on Intelligent Control Method of Camber for Medium and Heavy Plate Based on Machine Vision

**DOI:** 10.3390/ma18245668

**Published:** 2025-12-17

**Authors:** Chunyu He, Chunpo Yue, Zhong Zhao, Zhiqiang Wu, Zhijie Jiao

**Affiliations:** State Key Laboratory of Digital Steel, Northeastern University, Shenyang 110819, Chinazhaozhong@ral.neu.edu.cn (Z.Z.); wuzq@ral.neu.edu.cn (Z.W.)

**Keywords:** plate, camber, image processing, machine learning, feedback control

## Abstract

With the continuous development of intelligent manufacturing in the iron and steel industry, there are increasing requirements for the quality control and precision of steel products. Camber is one of the critical defects affecting product quality in medium and heavy plates. Its occurrence during the rolling process not only reduces the yield of plates but also leads to serious production accidents such as rolling scrap and equipment damage, increasing the operational costs of enterprises. Addressing the difficulties that camber is influenced by complex factors and direct modeling control is challenging, this study proposes a camber detection and control method for medium and heavy plates based on image processing and machine learning algorithms, relying on an actual plate production line. The Optuna-XGBoost model is used to mine and train the production data of plates rolling, extracting the optimal control experience of operators as the pre-control values for camber. The Optuna-XGBoost model achieves an *R*^2^ of 0.9999 on the training set and 0.9794 on the test set, demonstrating excellent fitting performance. Meanwhile, a camber detection technology during the plate rolling process is developed based on machine vision. A feedback control model for camber of medium and heavy plates based on distal lateral movement is established. The combined application of pre-control and feedback control reduces the occurrence of camber, ensuring the overall flatness of steel plates during the rolling process. This paper establishes an intelligent control framework for plate camber, synergized by data-driven pre-control and machine vision-based feedback control, offering a novel approach for the online optimal control of complex nonlinear industrial processes.

## 1. Introduction

Medium and heavy plates are among the most important steel products, widely used in various fields such as construction machinery, road and bridge engineering, shipbuilding, and the wind power industry [1]. Camber is a common shape defect during the rolling of medium and heavy plates, resulting from the uneven elongation along the length direction on both sides of the steel plate during rolling [2,3]. In the production process of medium and heavy plates, disturbances such as rolling mill parameters and environmental factors cause lateral asymmetric deformation of the rolled piece [4], leading to the occurrence of camber defects. Plate camber defects affect the dimensional accuracy and shape quality of the final product. In severe cases, they may even trigger production accidents such as tail flicking and steel piling [5], causing damage to production equipment, increasing the wear of rolling mill equipment, and raising the costs of equipment maintenance and enterprise operation.

Camber defect is one of the difficult problems that has long plagued the medium and heavy plate production. There are many influencing factors of camber, mainly involving rolling process [6], equipment factors, and rolled piece factors. Specific reasons include uneven temperature distribution [7], billet wedge shape [8,9], rolled piece lateral movement [10], roll lubrication [11], and difference in stiffness of the rolling mill, etc. The rolling piece factors and equipment factors influence each other, which contains a large number of nonlinear and coupling factors, making the formation mechanism of plate camber complex.

Currently, numerous scholars have conducted relevant research on the detection and control of camber in medium and heavy plates. Schausberger et al. [12,13,14] proposed an optimization-based camber control method for heavy plates by predicting the post-rolling plate profile through plate profile measurement and mathematical models. Prinz et al. [15] developed a feedforward control strategy for the plate lateral asymmetry under hot rolling conditions. Xu et al. [16] presented a camber control system based on the Takagi-Sugeno fuzzy model, which is capable of providing roll gap adjustment values. Radionov et al. [17] established a prediction method for roll gap asymmetry in mill stands, calculating the thickness difference based on the hydraulic cylinder pressure changes on the drive side and operation side of the mill to achieve compensation. Ding et al. [18] proposed a combination of a mathematical model for roll gap tilt adjustment and PSO-LSSVM-based camber prediction, which can provide tilt adjustment values for different products. Pietschnig et al. [19] developed an optimized feedforward controller and a Smith-predictive feedback controller based on a validated mathematical model, integrating them into a two-degree-of-freedom control model. Song et al. [20] proposed a camber control method integrating mechanism and data for hot-rolled plates, which was applied to a 1580 mm hot-rolled industrial production line and improved the camber control accuracy. Peng et al. [21] constructed a cluster analysis framework for camber in hot-rolled plates based on curve pattern recognition, achieving accurate classification of camber curve samples. Tong et al. [22] proposed an interpretable model for decoupling camber process parameters, using the LightGBM algorithm to model the camber prediction of rough-rolled intermediate billets and the SHAP method to enhance the model’s interpretability. Dong et al. [23] presented a camber prediction model for hot-rolled strips based on sequence-to-sequence learning with an attention mechanism, providing a new approach for the overall camber prediction of steel plates.

**Figure 1:**
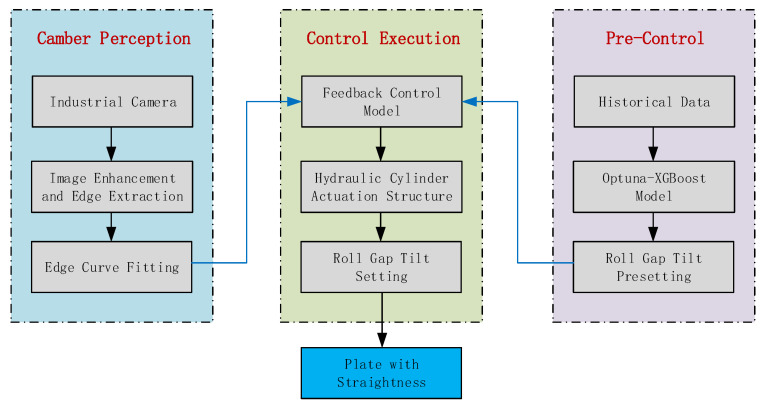
Overall framework diagram of the plate camber control system.

Camber is a complex technical challenge in steel production, involving equipment control across multiple processes [24]. Although certain progress has been made in camber control technology in recent years, the formation mechanism of camber is affected by the coupling effects of multiple factors due to the complexity of the plate rolling process [25], fully automated closed-loop control of camber has not yet been realized. Currently, camber control during plate production still relies primarily on manual experience for adjustments, which severely restricts the improvement of plate production efficiency. Taking the camber problem of a plate production line as the research background, this paper considers the complex production environment, adopts machine vision technology to establish a plate camber image detection system, integrates the production big data from the plate camber adjustment process, and constructs a pre-control and feedback control model for plate camber through machine learning algorithms. The overall framework diagram of the plate camber control system is shown in Figure 1. The machine learning-based pre-control model predicts the initial roll gap tilt adjustment by learning historical optimal operational experience, proactively offsetting camber that may be induced by common operating conditions. The feedback control model for distal displacement calculates the precise compensation adjustment based on the detected camber state of the plate, which is then superimposed with the pre-control value to achieve online rapid correction of the plate’s camber. Tests of the constructed model in actual production processes show that the camber control response is timely, the overall flatness of the steel plate is effectively guaranteed, and production efficiency and product yield are improved.

## 2. Machine Vision-Based Plate Camber Detection Method

Currently, the common adjustment method for camber in medium and heavy plates involves operators visually inspecting the formed shape of the steel plate to adjust the roll gap tilt amount on both sides. This approach is highly dependent on the operator’s work experience, involving significant subjective factors. Furthermore, the high on-site production speed makes it difficult to achieve timely control of camber. To meet the high real-time requirements for camber control in plate production, a targeted optimization algorithm has been developed based on traditional image processing techniques, enabling the extraction and fitting of the plate edges and centerline from images captured during the production process. In practical production, however, the images captured on-site suffer from blurriness due to the effects of light variations and residual water [26], which causes substantial interference to edge recognition. By employing image enhancement algorithms to process unstable images [27], stable edge information of plates can be obtained. This enhances the stability of image processing algorithms, eliminates on-site unstable factors, and provides camber bending information for the subsequent camber control model. Consequently, this research holds significant importance for the intellectualization of medium and heavy plate control systems.

The machine vision system developed in this study adopts two Basler monochrome area scan cameras with a resolution of 1280 × 1024 pixels, which are mounted above the roller table. Basler AG is headquartered in Ahrensburg, Germany. To adapt to the high-temperature and high-humidity environment of the rolling site, the cameras are equipped with high-temperature-resistant protective covers and water cooling systems.

### 2.1. Image Enhancement Algorithms

**Figure 2:**
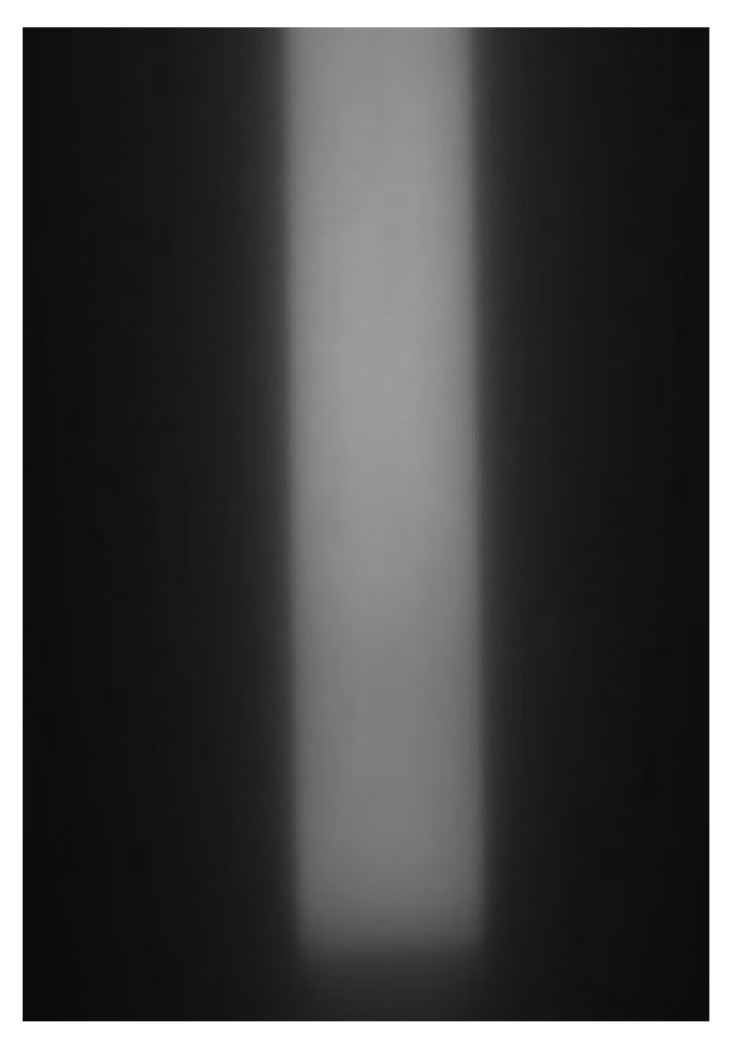
Overall low gray level of the plate image.

During the plate production process, image acquisition is affected by complex environmental factors. Specifically, the lighting conditions at the rolling site and the temperature variations in medium and heavy plates have a significant impact on image quality. Meanwhile, the rolling site suffers from insufficient lighting and uneven light distribution. In the later stages of multi-pass rolling, the temperature of plates gradually decreases, which in turn leads to a decline in image quality, and this phenomenon impairs the extraction of the plate edges, as shown in Figure 2.

**Figure 3:**
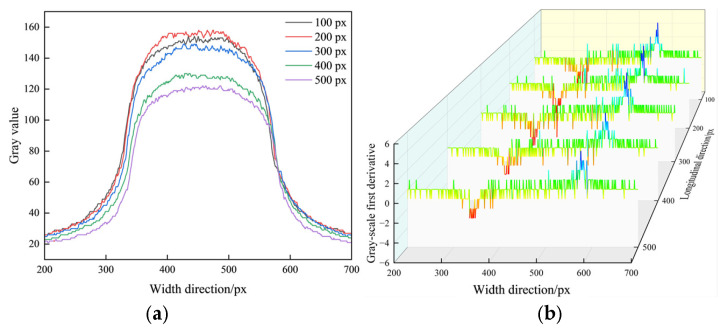
Statistical analysis of image grayscale variation: (**a**) image grayscale distribution; (**b**) first-order derivative variation in image grayscale (red and blue represent the regions with smaller and larger first-order derivatives, respectively, indicating the edge positions). The curves were generated via image processing implemented in Python 3.9 using the OpenCV and NumPy libraries.

Along the width direction of plate, the grayscale distribution and the variation in the first derivative of this row were statistically analyzed, as shown in Figure 3. In Figure 3a, the grayscale distribution of the acquired image exhibits obvious attenuation characteristics, which results in a decrease in image contrast. In the edge regions, the grayscale changes smoothly and slowly, the grayscale difference between the background and the edge parts narrows, and the signal-to-noise ratio (SNR) in the edge transition region declines. In Figure 3b, the first derivative value of the image’s grayscale gradient is excessively small, leading to the formation of continuous low-contrast bands in the edge transition region. Periodic grayscale fluctuations exist in the edge transition region, making it vulnerable to noise interference, and this causes significant disruption to the accurate localization and recognition of edges.

**Figure 4:**
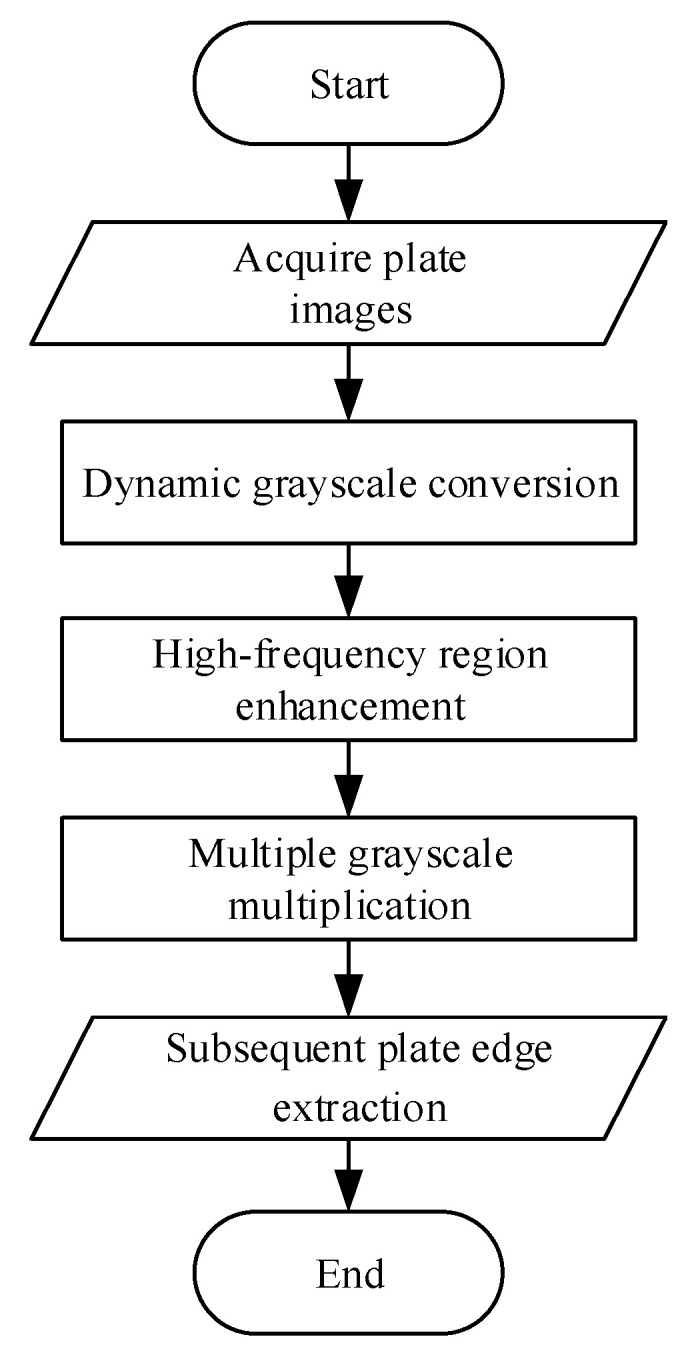
Flow chart of the grayscale enhancement algorithm.

To address the situation where excessively low overall grayscale values of the plate occur in subsequent rolling passes, which makes it difficult to distinguish the plate edges, processing the plate images with a grayscale enhancement algorithm can effectively resolve the problem of insufficient contrast in the edge transition regions and obtain clear and distinct edge contours. The overall workflow of the grayscale enhancement algorithm is illustrated in Figure 4.

Dynamic grayscale transformation tackles the issue of uneven grayscale distribution in images. It not only modifies the distribution range of grayscale values within the image but also increases the grayscale value of each pixel in the plate image, thereby effectively enhancing the overall contrast of the image. The high-frequency region enhancement algorithm first employs mean filtering to calculate the average grayscale value of the image within the window region, thereby performing smoothing processing on the image. Subsequently, it calculates the difference between the original grayscale value of the image and the average grayscale value through contrast enhancement, and multiplies this difference by a multiplicative influence factor to enhance the grayscale value of the image.

**Figure 5:**
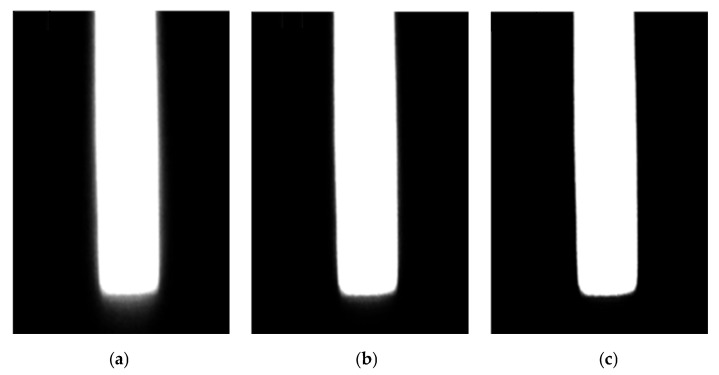
Effect of image grayscale value multiplication: (**a**) the first grayscale value multiplication; (**b**) the second grayscale value multiplication; (**c**) the third grayscale value multiplication.

After the image grayscale enhancement transformation is applied, the operation of multiplying image grayscale values can further enhance edge contrast. The images obtained after three consecutive grayscale value multiplication processes are shown in Figure 5. It can be observed that the grayscale difference between the foreground and background is gradually enlarged, while the grayscale values in the original edge transition regions of the image are gradually reduced, and this forms distinctly differentiated low-grayscale background regions and high-grayscale foreground regions, which facilitates the subsequent measurement of the plate’s camber. Naturally, excessive grayscale value multiplication operations on images will compromise edge precision. In practical applications, reasonable selections should be made based on the grayscale distribution of the images. Experimental results demonstrate that three iterations of gray-level multiplication, tailored to the brightness disparity between the hot plate and its background, achieves optimal edge enhancement performance. The associated edge loss is negligible, and the resulting high-clarity plate images provide a reliable foundation for subsequent measurement and processing workflows.

### 2.2. Caliper-Based Image Measurement Method

**Figure 6:**
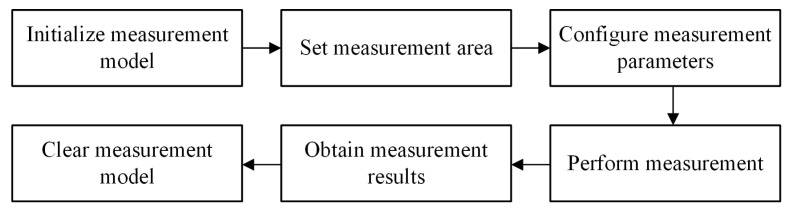
Flow chart of the caliper measurement method.

Caliper measurement is a sub-pixel level edge measurement algorithm, enabling high-precision edge information extraction in complex scenarios. Its principle is to set measurement regions and establish measurement models based on information such as the position and direction of the measured object in the image. After filtering via a Gaussian function, the algorithm determines the positions of sub-edges in the image according to grayscale threshold changes and edge polarity, boasting high measurement accuracy. The specific measurement process is shown in Figure 6.

**Figure 7:**
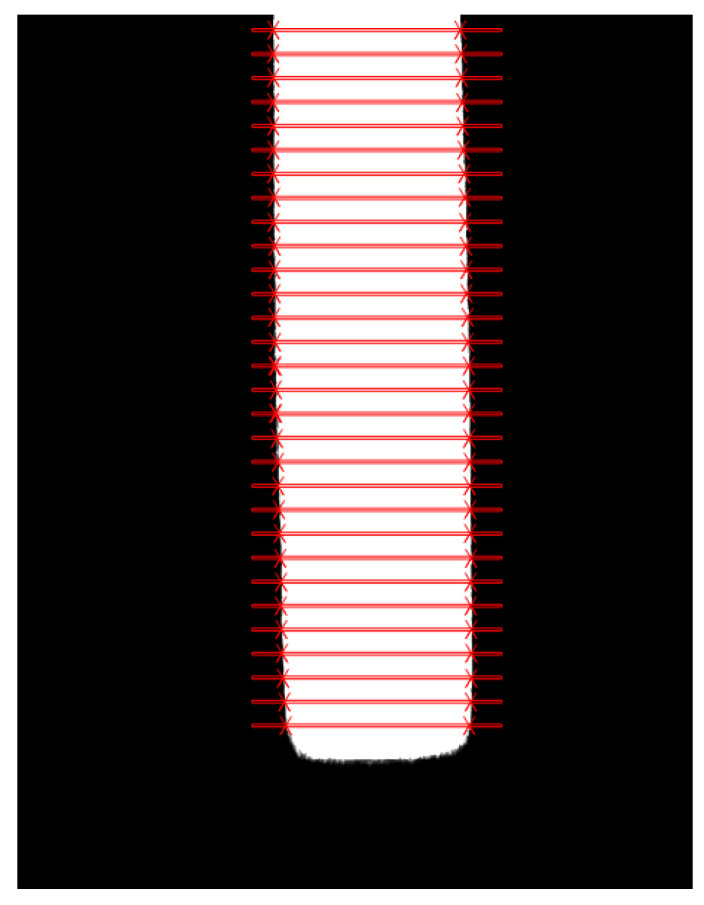
Extract edge points diagram (red lines indicate the detected positions of the plate edges).

In practical applications, multiple measurement positions are generated along the longitudinal direction of the plate based on its location, and the caliper measurement algorithm is used to measure the edge information of the plate at designated positions. The algorithm detects the accurate information of the edges on both sides of the plate according to the changes in grayscale values at the detection positions. The schematic diagram of plate edge detection based on the caliper measurement algorithm is shown in Figure 7. It can be seen that the algorithm accurately identifies the coordinate points of the edges on both sides of the medium and heavy plate.

### 2.3. Obtaining the Plate Camber Value via the Least Squares Method

The least squares method can be used to calculate the best-fit curve in data fitting. Its core idea is to minimize the sum of squared errors between the fitted results and the actual results [28], thereby ensuring the accuracy of the fitting. After obtaining the coordinate information of the edge points on both sides of the plate, the least squares method is used to fit the quadratic curves of the edges and the center line of the plate. Assuming that the quadratic equation of the fitted curve is $y=ax^{2}+bx+c$, then the error function *L* between the actual coordinate point $\left(x_{i},y_{i}\right)$ and the fitted curve can be expressed as:

(1) $$L={\sum_{i=1}^{N}\left[y_{i}-\left(ax_{i}^{2}+bx_{i}+c\right)\right]}^{2}$$

In the formula, a, b and c are the coefficients of the fitted quadratic curve; *N* is the number of coordinate points.

By taking the partial derivatives of the error function with respect to a, b, and c, setting them equal to zero, and rearranging the results, we obtain Equation (2), which further allows us to solve for the coefficients of the quadratic curve.

(2) $$\left[\begin{array}{l} \sum x_{i}^{2}y_{i}\\ \sum x_{i}y_{i}\\ \sum y_{i} \end{array}\right]=\left[\begin{array}{l} \sum x_{i}^{4}\;\;\;\;\;\;\sum x_{i}^{3}\;\;\;\;\;\;\sum x_{i}^{2}\\ \sum x_{i}^{3}\;\;\;\;\;\;\sum x_{i}^{2}\;\;\;\;\;\;\sum x_{i}\\ \sum x_{i}^{2}\;\;\;\;\;\;\sum x_{i}\;\;\;\;\;\;\;\;\;\;\;n \end{array}\right]\left[\begin{array}{l} a\\ b\\ c \end{array}\right]$$

**Figure 8:**
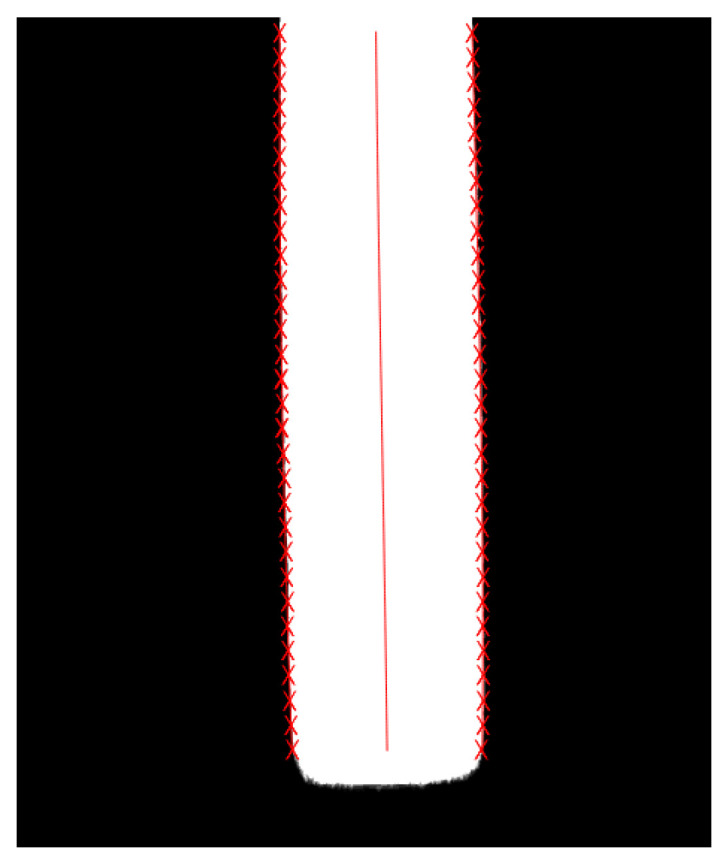
Edge and center line positions fitted by the least squares method (red crosses represent the identified edge points; the red line denotes the fitted curve of the steel plate center).

The position information of the edges and center line obtained through quadratic curve fitting via the least squares method is shown in Figure 8. The acquired coordinate points are fitted using the least squares method to generate a smooth curve [29], which can reflect the real trend of the edges and center line of the plate, thus improving the accuracy of image detection.

### 2.4. Outlier Coordinate Point Rejection Algorithm

**Figure 9:**
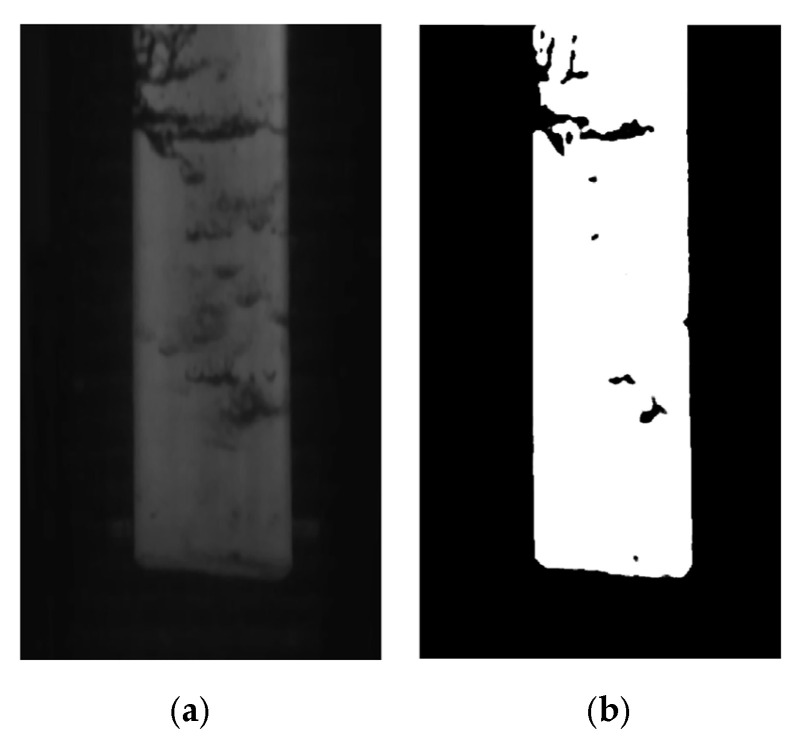
Before and after gray scale enhancement of an image with residual water: (**a**) before image grayscale enhancement; (**b**) after image grayscale enhancement.

In the actual production process of medium and heavy plates, the water residue problem caused by the high-pressure water descaling process will inevitably lead to defects such as blurriness and noise in the acquired images, resulting in a significant degradation of image quality, as shown in Figure 9a. The flow of residual water on the slab surface causes motion blur areas and noise interference, leading to significant grayscale attenuation in local regions. This makes the grayscale values of the real plate edge parts too low, resulting in the failure of edge detection. After grayscale enhancement processing, as shown in Figure 9b, the grayscale of most areas in the foreground of the image is effectively enhanced. However, the parts with excessively low grayscale values will become 0, which causes continuous or discontinuous voids inside and at the plate edges. The original real edge information is omitted during the image processing, making it impossible to calculate the original and true edge position information.

**Figure 10:**
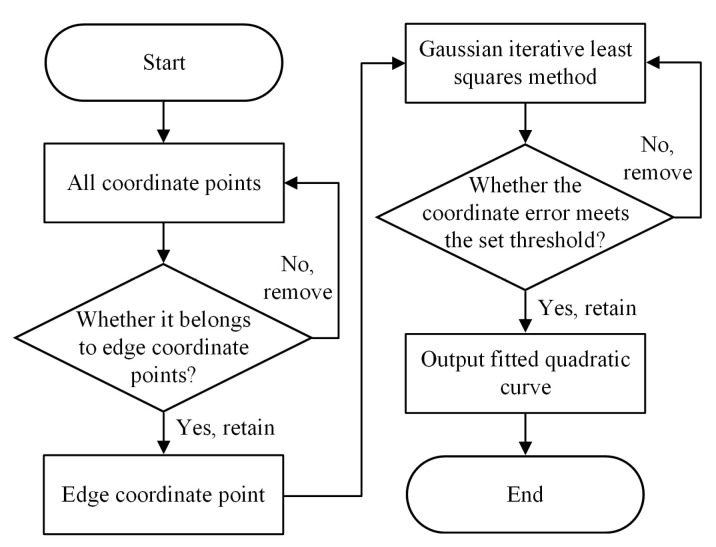
Flow chart of abnormal coordinate point rejection algorithm.

To address the problem of plate being obscured by water vapor during the rolling process, the iterative reweighted least squares method based on the Gaussian kernel function is employed to fit the plate edge position information. Abnormal detected edge coordinate points are removed, thus enhancing the stability of identifying the plate edge position information. The flow of the abnormal coordinate point removal algorithm is shown in Figure 10.

The iterative reweighted least squares method based on the Gaussian kernel function is a highly effective curve fitting method. The use of the Gaussian kernel function enables the fitted curve to adjust weight values according to the relative distance between coordinate points and the curve, reducing the impact of outlier coordinate points on the fitting results [30]. The iterative weighting process can dynamically update valid coordinate points until all coordinate points meet the requirements or the maximum number of iterations is reached, and finally output the fitted curve. In this process, the impact of abnormal points is effectively reduced, and the accuracy of curve fitting is improved. The iteration process of the iterative reweighted least squares method based on the Gaussian kernel function is as follows:

(1) Construction of the Gaussian weight matrix

For the given set of data points {*x_i_*}, the Gaussian kernel function is calculated as follows:

(3) $$K(x_{i},x_{j})=\frac{1}{\sqrt{2\pi }\sigma }e^{-\frac{{\left(x_{i}-x_{j}\right)}^{2}}{2\sigma^{2}}}$$

Equation (3) defines the Gaussian kernel function, where $K(x_{i},x_{j})$ denotes the similarity between samples $x_{i}$ and $x_{j}$, and *e* is the natural constant, *σ* represents the kernel bandwidth parameter, and $\sqrt{2\pi }\sigma$ is the normalization factor of the Gaussian distribution.

The weight matrix is constructed according to Equation (4), where N denotes the total number of input samples; $W_{ij}$ is an element of the Gaussian kernel function $K(x_{i},x_{j})$, and it quantifies the similarity between the *i*-th sample $x_{i}$ and the *j*-th sample $x_{j}$. Transform the matrix $W_{ij}$ into the diagonal matrix $W_{diag}$, whose diagonal elements are computed by summing the columns of $W_{ij}$.

(4) $$W_{diag}\left(i,j\right)=\sum_{i=1}^{N}W_{ij}$$

In the formula, the diagonal elements represent the comprehensive weight of the corresponding data point, and the greater the weight, the greater the influence of this point.

(2) Computation of the weighted coefficients

By solving the linear system of equations $\alpha ={\left(\left(X^{T}W_{diag}X\right)\right)}^{-1}X^{T}W_{diag}y$, the least squares curve coefficient α is obtained.

(3) Update of the valid data points

Based on the prediction residuals of the current model, filter the valid coordinate points, calculate all predicted values ${\hat{y}}_{i}$, compare them with the actual values $y_{i}$, and compute the residual $e_{i}=y_{i}-{\hat{y}}_{i}$. According to the set residual threshold *δ*, retain the data points that satisfy $\left|e_{i}\right|\leq \delta$ and exclude the outlier coordinate points.

(4) Iteration process

Input the initial set of data points and the number of iterations, then repeat the aforementioned steps (1)–(3) until the number of iterations is reached or the error converges, and finally output the fitted curve.

**Figure 11:**
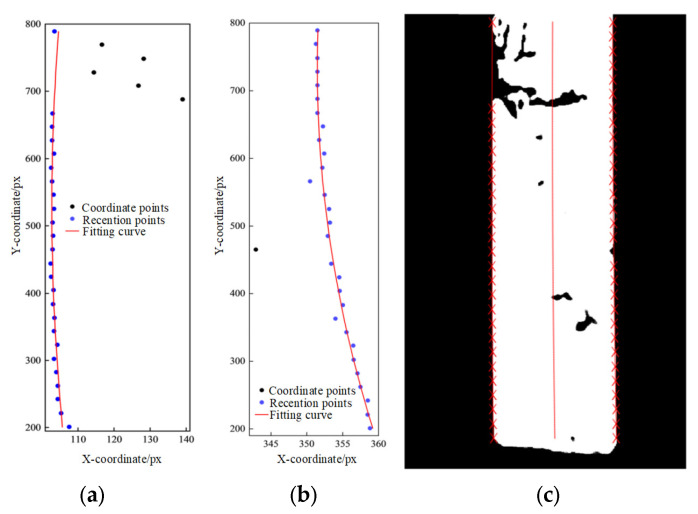
Gaussian kernel function based iterative weighted least squares fitting effect plot: (**a**) the set of coordinate points on the left side of the plate; (**b**) the set of coordinate points on the right side of the plate; (**c**) final fitting effect of the plate camber curve (red crosses represent the identified edge points; the red line denotes the fitted curve of the steel plate center).

The contour edge information of steel plates typically exhibits a trend of continuous change. The iterative reweighted least squares method based on the Gaussian kernel function can eliminate outlier coordinate points [31], capture the variation trend of edge contours, and restore the true plate edge trend. The fitted edge and central curves are shown in Figure 11.

## 3. Machine Learning-Based Pre-Control Model for Plate Camber

In the production process of medium and heavy plates, various factors such as uneven temperature distribution of plates, wedge-shaped billets, roll wear, and differences in stiffness between the two sides of the mill can all cause camber defects. These factors exhibit nonlinear and strong coupling characteristics, making it difficult for traditional models to conduct effective analysis. During the plate rolling process, operators evaluate relevant information including the current camber condition of the plates, the mill roll system, and rolling process parameters. They proactively determine the roll gap tilt setting of the mill, and relevant data collected in the rolling system form the pre-control operational experience for medium and heavy plate camber.

### 3.1. Data Processing

#### 3.1.1. Data Selection and Preprocessing

The dataset used is derived from a 3500 mm plate production line, including relevant parameters during the plate rolling process such as zero position difference, rolling speed, inlet and outlet thicknesses, workpiece width and length, total rolling force, workpiece temperature, and camber value, as shown in Table 1. We extracted continuous rolling process data covering a 2-month normal production cycle from the production database, ensuring strict correspondence between the data and production events through a timestamp synchronization mechanism. After data cleaning and preprocessing, the final valid dataset used for model construction contains 15,860 complete rolling pass samples. The input variables are mainly the rolling process parameters and the camber value, while the output is the tilt adjustment amount. Since the upper work roll number and lower work roll number in the data are strings, the character values of the upper and lower work rolls are converted to numerical values and input into the model for modeling.

The production data of medium and heavy plates is characterized by complex dimensions, diverse types, and large scale. The collected data is often affected by equipment abnormalities, sensor failures, or sudden factors, resulting in deviations in data quality. Such data not only affects data reliability but also impairs the predictive performance of machine learning models. Therefore, it is necessary to remove missing values, duplicate values, and special values in the dataset to eliminate the impact of outliers on decision-making. Meanwhile, normalization should be performed before data training. Min-max normalization is adopted to map the converted data to the interval [0, 1], and the conversion function is given by Equation (5), where $x_{\min}$ denotes the minimum value in the data, and $x_{\max}$ denotes the maximum value in the data.

(5) $$x^{*}=\frac{x-x_{\min}}{x_{\max}-x_{\min}}$$

#### 3.1.2. Clustering Algorithm

The Mean Shift [32] algorithm is a density-based non-parametric clustering algorithm, which has good adaptability to irregular and complex-shaped data. The basic idea is to assume that datasets of different clusters have different probability density distributions. By performing density estimation on local data points, the direction of the fastest increase in data point density is found. Through an iterative manner, the local cluster centers are continuously moved towards the direction of increasing data point density in the local region until they converge to the local maximum density points.

Suppose there are n data points $\left\{x_{1},x_{2},x_{3},\ldots ,x_{i}\right\}$, where each $x_{i}\in R^{d}$. A window is used to determine the neighborhood size of the data points, then the estimated density function for a certain data point is as follows:

(6) $$\hat{f}(x)=\frac{1}{nh^{d}}\sum_{i=1}^{n}K(\frac{x-x_{i}}{h}),$$

where $\hat{f}(x)$ denotes the kernel density estimate at the target point x; n denotes the total number of data points; d denotes the feature dimension; *h* is the bandwidth; $K(x)=k\left({\left\|x\right\|}^{2}\right)$ is the kernel function.

By introducing the kernel function and weight coefficients into the calculation of the mean vector, the Mean Shift vector is as follows:

(7) $$M_{h}(x)=\frac{\sum_{i=1}^{n}G_{H}(x-x_{i})w(x_{i})(x_{i}-x)}{\sum_{i=1}^{n}G_{H}(x-x_{i})w(x_{i})},$$

where $G_{H}$ is the scaled kernel function based on the bandwidth matrix H; $w(x_{i})$ is the weight matrix of sample points in the high-dimensional spherical neighborhood, and *H* is the bandwidth matrix, which represents a symmetric positive definite matrix proportional to the identity matrix.

This paper adopts the Mean Shift algorithm for data clustering processing, eliminates data points with large deviation, which can further improve the data quality and generate the optimal plate camber adjustment dataset.

### 3.2. Data Training Algorithm

#### 3.2.1. Support Vector Regression

Support Vector Machine (SVM), proposed by Vapnik et al. [33] in 1982, is a machine learning algorithm for regression. It is constructed based on the VC (Vapnik-Chervonenkis) dimension theory in statistical learning theory and the principle of structural risk minimization. Support vector regression (SVR) finds the optimal hyperplane in the feature space to minimize the distance from most data points to the hyperplane [34]. It allows a certain error margin *ε* between predicted values and actual values [35], making the dense regions of data points distribute as much as possible within the band interval to achieve optimal fitting, this characteristic confers support vector regression with a certain level of robustness.

#### 3.2.2. Decision Tree

Decision Tree (DT) is a classic tree-structured machine learning algorithm [36]. It constructs a tree recursively based on the features of input data, and after pruning, yields the final predictive model. First, DT partitions sample data into distinct regions; subsequently, predictions for target values are made at each leaf node. At each internal node, the data is split into subsets according to feature values [37], with predictions based on the mean value of each subset. As the stepwise partitioning proceeds, prediction errors are gradually reduced.

#### 3.2.3. Random Forest

Random Forest (RF) is an ensemble learning model that makes predictions by constructing multiple decision trees [38]. Decision trees perform data sampling via the bootstrap sampling strategy: by sampling with replacement from the original data, multiple distinct training subsets are generated [39]. During the construction of decision trees, in each node splitting, a subset of features is randomly selected from all features. By integrating multiple models into a strong learner, the bias of a single model is reduced [40], and the model’s accuracy is enhanced.

#### 3.2.4. Extreme Gradient Boosting

Extreme Gradient Boosting (XGBoost), proposed by Chen et al., is a gradient boosting-based ensemble learning algorithm for solving classification and regression problems in machine learning [41]. The core of XGBoost lies in constructing multiple decision tree models during the training iteration process and gradually improving model accuracy through gradient boosting to minimize the objective function [42]. In each iteration, the XGBoost algorithm employs the forward stagewise algorithm for greedy learning during training and constructs new decision trees based on the residuals between the predicted results of decision trees in the current model and the actual values. It continuously reduces these residuals to enhance the model’s predictive performance and accuracy.

#### 3.2.5. Optuna Framework

A major challenge for machine learning models during training is hyperparameter optimization [43]. The optimization process must balance computational cost and time cost, and the selection of hyperparameters is crucial to the model’s performance on the dataset. Hyperparameter selection in machine learning models is a time-consuming and labor-intensive process [44]. When the model needs to select multiple hyperparameters, the number of hyperparameter combinations exhibits exponential growth [45], which greatly increases the difficulty of finding the optimal hyperparameters and makes it difficult to meet the time cost and computational resource requirements.

Optuna is an efficient automated hyperparameter optimization software characterized by being lightweight [46], easy to parallelize, user-friendly, easy to integrate, and equipped with visualization capabilities. Its core goal is to efficiently find the optimal hyperparameter combinations for machine learning models [47], which is mainly reflected in the sampling and pruning processes. The sampling method adopted is the Tree-structured Parzen Estimator, which can dynamically generate new hyperparameter combinations based on the generated evaluation results. It tends to move towards relatively optimal hyperparameter regions for more meaningful exploration. The pruning mechanism enables multiple hyperparameter combinations to run simultaneously. When suboptimal results are identified based on the objective function, it stops these combinations early to save computational resources [48].

**Figure 12:**
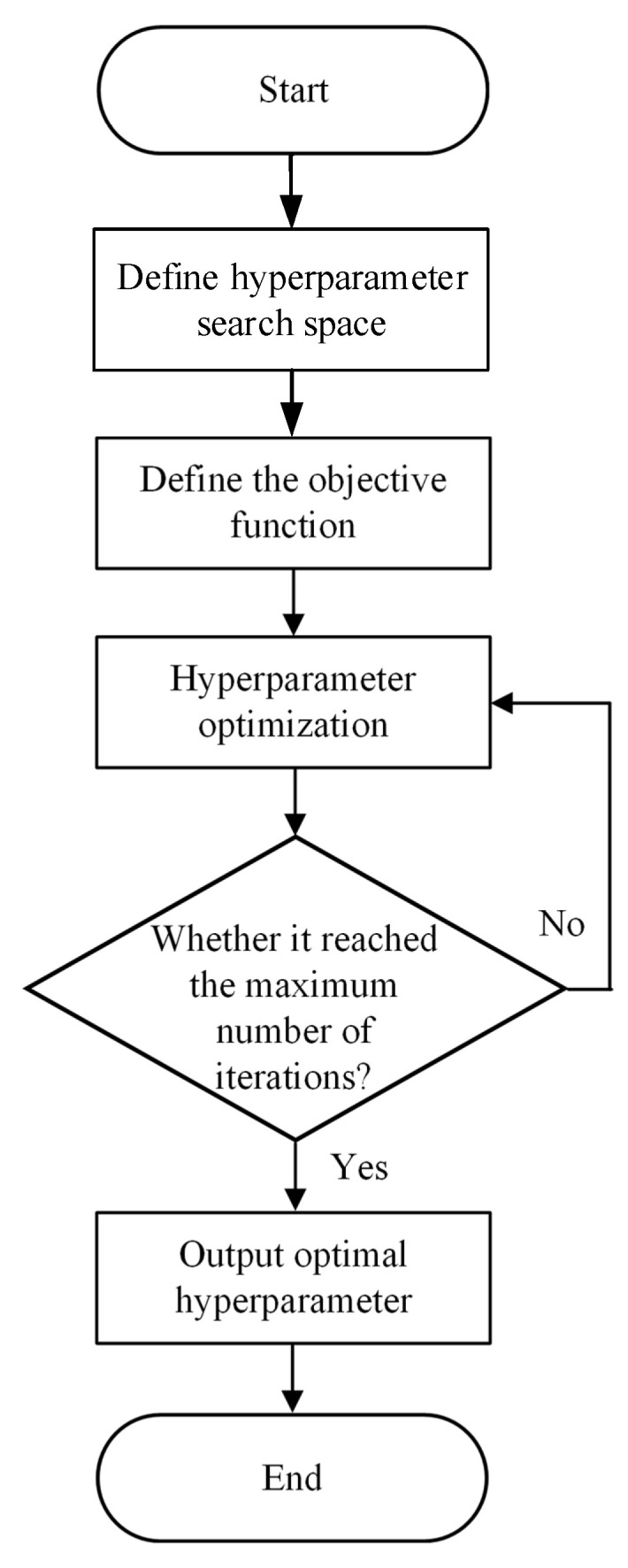
Optuna hyperparameter optimization flowchart.

The steps for optimizing model hyperparameters using Optuna include defining the model’s hyperparameter search space, specifying the model’s objective function, setting the number of trials, and acquiring the model’s optimal hyperparameters and corresponding objective function value. The Optuna hyperparameter optimization process is illustrated in Figure 12.

#### 3.2.6. Metric Evaluation

This paper focuses primarily on the difference between predicted values and actual values. To better measure the fitting performance of the constructed models, four commonly used performance metrics in predictive models are employed to evaluate model performance: coefficient of determination (*R*^2^), mean squared error (*MSE*), root mean squared error (*RMSE*), and mean absolute error (*MAE*).

(8) $$R^{2}=1-\frac{\sum {\left(y_{i}-{\hat{y}}_{i}\right)}^{2}}{\sum {\left(y_{i}-{\bar{y}}_{i}\right)}^{2}}$$

(9) $$RMSE=\sqrt{\frac{1}{N}\sum_{i=1}^{N}{\left(y_{i}-{\hat{y}}_{i}\right)}^{2}}$$

(10) $$MSE=\frac{1}{N}\sum_{i=1}^{N}{\left(y_{i}-{\hat{y}}_{i}\right)}^{2}$$

(11) $$MAE=\frac{1}{N}\sum_{i=1}^{N}\left|y_{i}-{\hat{y}}_{i}\right|$$

In the formula, *N* denotes the total number of actual values in the sample data, $y_{i}$ represents the *i*-th actual value, ${\hat{y}}_{i}$ is the *i*-th predicted value, and ${\bar{y}}_{i}$ stands for the mean of all actual values.

### 3.3. Analysis of Experimental Results

#### 3.3.1. Data Training Settings

**Figure 13:**
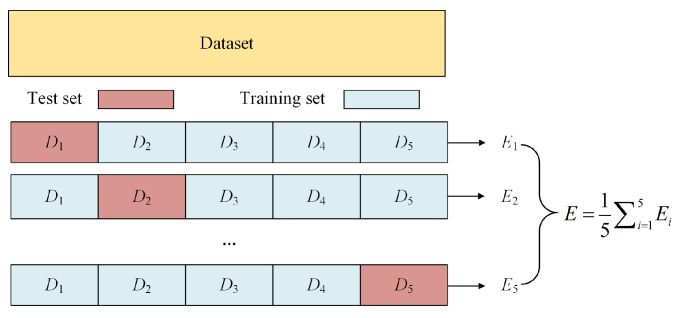
Schematic diagram of cross-validation.

In this paper, the sample dataset is divided into a training set and a test set in a 4:1 ratio. The objective function of Optuna adopts the average MSE of 5-fold cross-validation [49]. Using this objective function can ensure the model exhibits excellent generalization ability across different data subsets, thereby enhancing the model’s stability and reducing the risk of overfitting. The number of trials is set to 100 to maximize the chance of finding the optimal hyperparameter combination. 5-fold cross-validation is illustrated in Figure 13. The versions of the used environment are shown in Table 2.

#### 3.3.2. Hyperparameter Optimization Process

**Figure 14:**
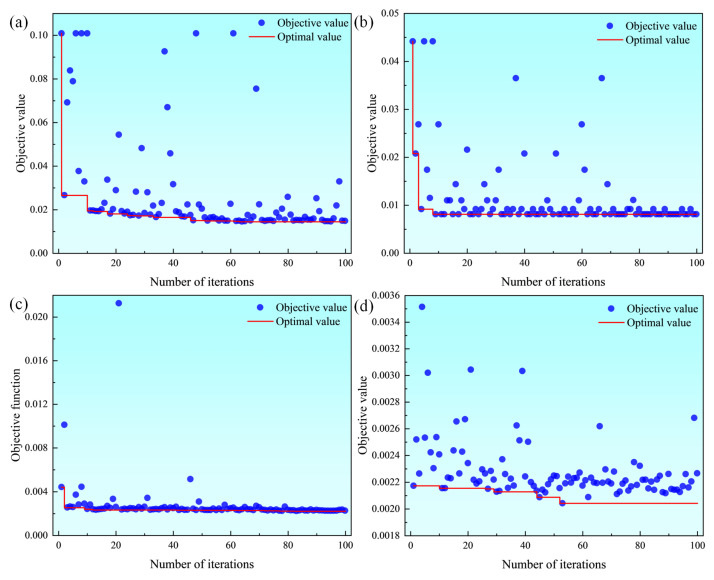
Hyperparameter optimization history plot: (**a**) support vector regression; (**b**) decision tree; (**c**) random forest; (**d**) extreme gradient boosting.

The Optuna framework provides visualization tools to facilitate understanding of the impact of hyperparameters on model performance and the importance of hyperparameters in the model. Figure 14 presents the hyperparameter optimization history plot. It displays the changes in the objective function over 100 iterations for different models under the Optuna framework. The scatter points represent the objective function values during iterations, and the red curve represents the optimal objective function values.

In Figure 14a,b, the support vector regression and decision tree models have high objective function values in the initial stage of the trials. This indicates that the error of the initial hyperparameter combinations is relatively high. As the number of trials increases, the objective function values decrease rapidly. This demonstrates that the Optuna framework improves model performance by adjusting hyperparameters. In the later stage of the trials, the objective function values gradually stabilize. This shows that a relatively ideal set of hyperparameters has been found and the model performance has reached the optimal level. The support vector regression and decision tree models achieve their minimum objective function values in the 64th and 8th iterations, which are 0.01447 and 0.00813, respectively. In Figure 14c,d, the random forest and extreme gradient boosting models show a slow decline during the iteration process. This indicates that the model performance gradually improves with Optuna optimization. The random forest and extreme gradient boosting models obtain their minimum objective function values in the 88th and 53rd trials, which are 0.00221 and 0.00204, respectively.

**Figure 15:**
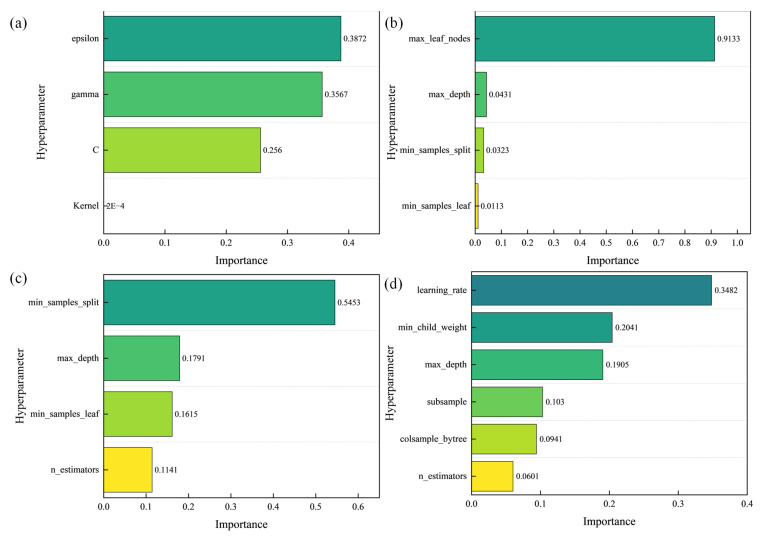
Hyperparameter importance schematic plot: (**a**) support vector regression; (**b**) decision tree; (**c**) random forest; (**d**) extreme gradient boosting.

Figure 15 presents the importance scores of each hyperparameter, which are automatically calculated by the Optuna framework through analyzing historical trial records during the training process. Analyzing the hyperparameter importance plot can guide model optimization and facilitate targeted adjustments of critical hyperparameters. The most important hyperparameters for the support vector machine, decision tree, and random forest models are, respectively, as follows: C (penalty coefficient), gamma (radial basis function kernel), epsilon (insensitive loss coefficient), kernel (kernel function selection), max_leaf_nodes, and min_samples_split, etc. For the XGBoost model, the most critical hyperparameter is learning_rate. The hyperparameter ranges of the models and the optimal hyperparameters after optimization using Optuna are presented in Table 3.

#### 3.3.3. Results Comparison

**Figure 16:**
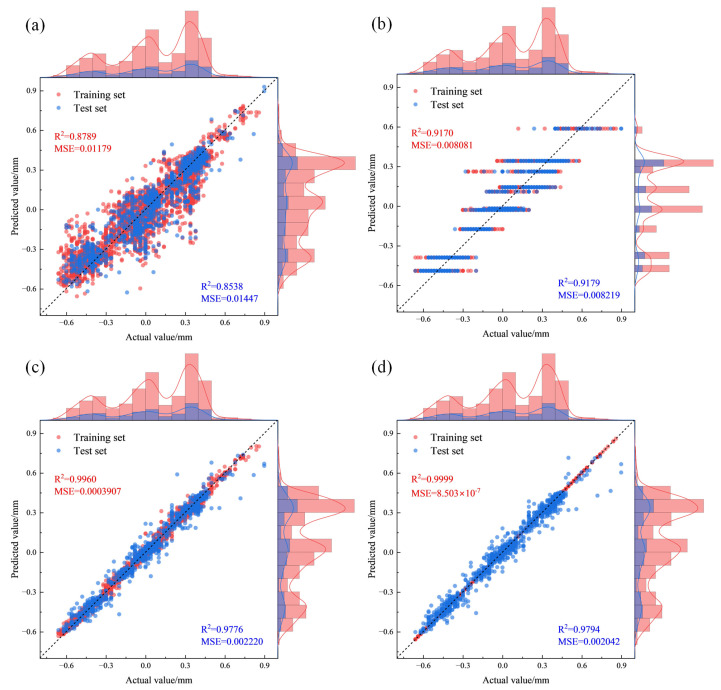
Predictive results scatter plot: (**a**) support vector regression; (**b**) decision tree; (**c**) random forest; (**d**) extreme gradient boosting.

Figure 16 presents the predictive performance of various models on the training set and test set. Herein, the red data represent the predictive results of the training set, and the blue data represent those of the test set. The histogram illustrates the data distribution on the corresponding axis.

The data scatter points of the support vector machine model are relatively scattered with a significant degree of deviation. It exhibits the worst fitting performance on both the training set and test set. On the training set, it has an *R*^2^ of 0.8789 and an MSE of 0.01179, while on the test set, the *R*^2^ and MSE are 0.8538 and 0.01447, respectively.

For the decision tree model, the data distribution on the predicted value axis is narrow and uneven. Its data scatter points exhibit a multi-linear distribution with a relatively large degree of deviation. On the training set, its *R*^2^ and MSE are 0.9170 and 0.00808, while on the test set, the values are 0.9179 and 0.00813. Its fitting performance is superior to that of the support vector machine, with a lower MSE.

The data points of the random forest and XGBoost models are concentrated near the diagonal line, showing excellent fitting results on both the training and test sets. On the training set, the random forest model has an *R*^2^ of 0.9960 and an MSE of 0.000391, while the XGBoost model has an *R*^2^ of 0.9999 and an MSE of 8.503 × 10^−7^. On the test set, the random forest model achieves an *R*^2^ of 0.9776 and an MSE of 0.00222, whereas the XGBoost model reaches an *R*^2^ of 0.9794 and an MSE of 0.00204. The XGBoost model outperforms the random forest on both sets and demonstrates the most excellent fitting performance.

The regression evaluation metrics of different machine learning models after hyperparameter optimization via Optuna, on both the training set and test set, are shown in Table 4. By comparing these results, it can be concluded that the Optuna-XGBoost model exhibits outstanding performance in predicting the roll gap tilt value for the plate camber. The model demonstrates strong predictive performance on both the training and test sets, indicating excellent generalization ability.

Analysis of data characteristics during model training indicates that the dataset contains a small number of high-target-value samples corresponding to non-ideal process states, resulting in relatively compromised prediction accuracy within this range. A key optimization direction lies in adjusting the data structure through sample augmentation and distribution balancing, thereby significantly enhancing the model’s generalization ability and robustness, and providing reliable support for accurate prediction in practical industrial scenarios.

#### 3.3.4. Feature Importance Ranking

**Figure 17:**
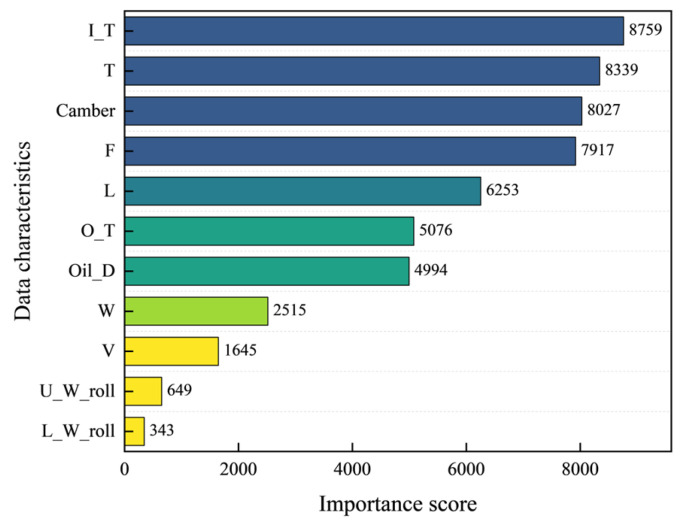
Characteristic importance chart.

Feature importance scores represent the value of data features in the construction of the model’s decision-making process, serving as a key metric for measuring the contribution of features in the model. Taking the optimal XGBoost model with hyperparameters optimized by Optuna as the benchmark, we evaluate the importance of each feature by calculating two metrics: gain-based feature importance and cover-based feature importance during the decision tree splitting process. The importance scores of various data features are presented in Figure 17. There are significant differences in the importance contributions of different data features to the tilt adjustment amount, and the ranking of importance scores provides a basis for adjusting the tilt adjustment amount. Among these features, the entry thickness, temperature, and camber of the workpiece have relatively high importance scores, indicating that the tilt adjustment amount is closely related to these factors.

The entry thickness serves as a critical geometric factor determining the distribution of reduction ratio across the plate cross-section. Thickness discrepancies between the two sides directly induce uneven longitudinal elongation of the plate during rolling, which constitutes one of the primary causes of camber formation. Non-uniform temperature distribution across the plate cross-section significantly affects the metal’s deformation resistance: the side with higher temperature exhibits lower deformation resistance, leading to greater elongation under the same rolling force and thereby driving the plate to bend toward the cooler side. The actual measured value of camber serves as the most direct feedback signal. The control model must determine the required corrective tilt adjustment based on the observed magnitude and direction of the plate’s camber. The Optuna-XGBoost model extracts effective predictive features from the data, and its feature importance ranking accurately corresponds to the key factors influencing plate camber. This indicates that the data-driven model can capture and reflect the intrinsic mechanisms of the rolling process.

## 4. Feedback Control Model for Plate Camber

Traditional camber detection and control methods rely on manual visual inspection and empirical adjustment, suffering from significant lag and low precision. Such drawbacks make these methods inadequate for the high-efficiency rolling control requirements of modern production lines. This study develops a machine vision-based intelligent control system for plate camber. It extracts the plate centerline via image detection to obtain the lateral offset of camber, and performs adjustments based on the camber control strategy for medium and heavy plates [50], ensuring the flatness of subsequent plates. Feedback control adjusts based on the preset roll gap tilt value output by the pre-control model. This design significantly shortens the response time of feedback control and reduces the required adjustment range, thereby improving the control efficiency and stability of the entire system.

### 4.1. Plate Far-End Lateral Displacement Detection Method

The plate length is extended with the increase in rolling passes, and the amplification effect caused by far-end lateral displacement makes the camber more significant. The far-end displacement effect will further amplify the camber defects, and improving the detection accuracy of the image detection system for camber.

**Figure 18:**
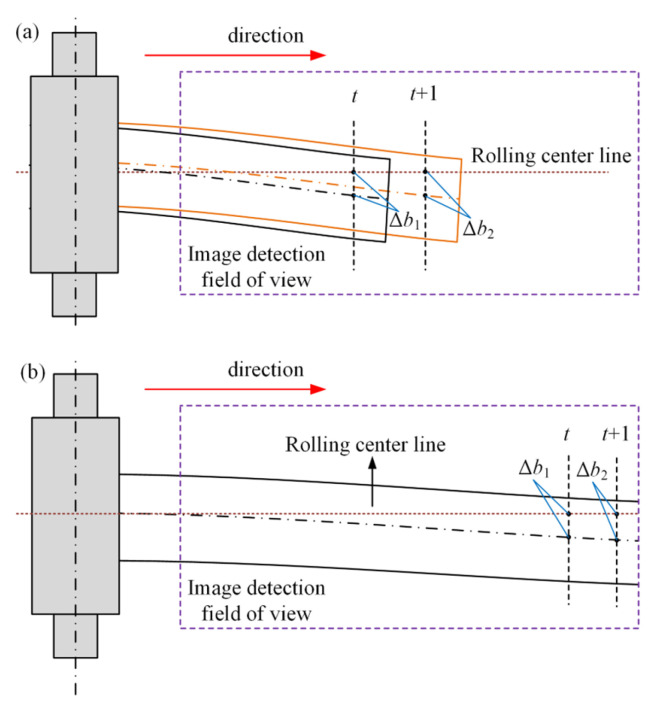
Schematic diagram of camber distal traverse detection: (**a**) plate head is within the field of view (The solid black line represents the position of the plate at time *t*, and the dashed black line represents the center line of the plate at time *t*; the solid orange line represents the position of the plate at time *t* + 1, and the dashed orange line represents the center line of the plate at time *t* + 1); (**b**) plate head is outside the field of view.

The far-end lateral displacement detection of plate is based on machine vision technology to detect the lateral offset between the centerline of the front part of the rolled plate and the centerline of the rolling mill, thereby determining the camber condition. As shown in Figure 18, it directly reflects the camber state along the rolling direction. When the head of the plate is within the field of view of the image detection system, the stable region close to the head is chosen to avoid misjudgment in detection, which is prone to be caused by the irregularly deformed region at the plate head. Compared with the head region, the stable region near the head of the plate has more uniform deformation and can better represent the overall camber condition of the plate. When the head of the plate exceeds the field of view of the image detection system, the stable region of the plate at the farthest field of view is selected as the detection object. This can utilize the far-end amplification effect to improve the camber detection accuracy, thereby enhancing the detection stability of the plate camber.

### 4.2. Analyzing the Speed Difference on Both Sides of the Workpiece

The formation of camber in medium and heavy plates can be simplified as follows: during the rolling process, the different elongation ratios on both sides of the steel plate cause the plate to undergo rigid body rotation around a certain point within a time interval Δt. This rotation accumulates continuously, thereby generating the camber phenomenon.

**Figure 19:**
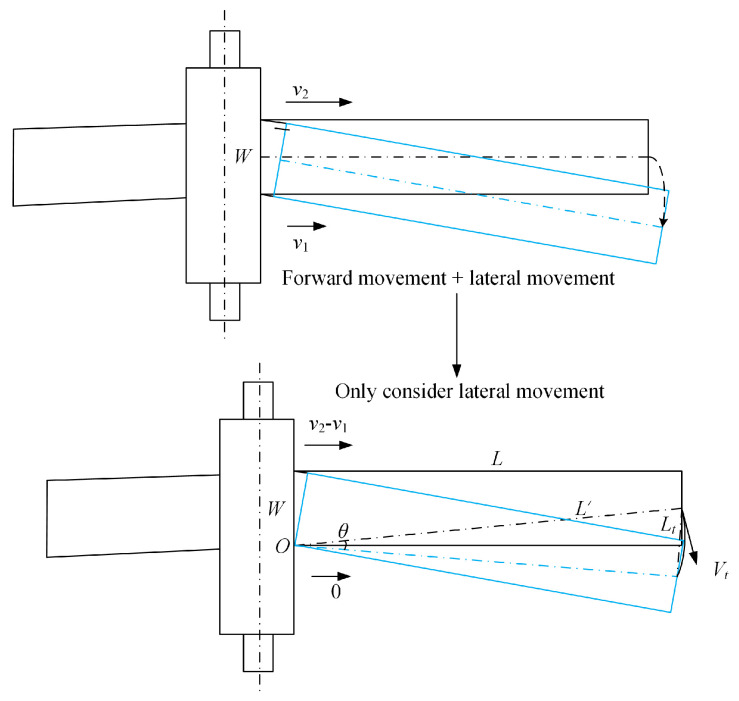
Schematic diagram of rolled rigid body rotating camber.

Taking the work rolls as rigid rolls, we calculate the speed difference on both sides of the workpiece within a time interval Δt during the rolling process, as shown in Figure 19. The speeds on the two sides of the workpiece are denoted as $v_{1}$ and $v_{2}$ (where $v_{2}>v_{1}$), and $v_{2}$ is expressed as $v_{1}+\Delta v$.

Assuming the workpiece is a rigid body as a whole, it rotates around point *O*. The width of the workpiece is W, and its rigid body rotation angle is *θ*. The lateral displacement of the workpiece within time Δt is $L_{t}$, so the relationship between the rotation angle and the lateral displacement can be derived as follows:

(12) $$\sin(\frac{\Delta \theta }{2})=\frac{L_{t}/2}{L^{\prime }},$$

where $L^{\prime }=\sqrt{{\left(\frac{W}{2}\right)}^{2}+L^{2}}$; *L* denotes the length of the workpiece.

After the transformation of Equation (12), the rotation angle of the workpiece, denoted as Δ*θ*, can be obtained as follows:

(13) $$\Delta \theta =2\arcsin(\frac{L_{t}/2}{L^{\prime }})$$

Based on $\frac{\Delta \theta }{\omega }=\Delta t$ and $\omega =\frac{V_{t}}{L^{\prime }}$, the following can be obtained:

(14) $$\frac{\Delta v}{W}=\frac{\Delta \theta }{\Delta t}$$

(15) $$\Delta v=\frac{\Delta \theta \cdot W}{\Delta t}=\frac{2\arcsin(\frac{L_{t}/2}{L^{\prime }})\cdot W}{\Delta t}$$

where Δv denotes the velocity difference between both edges of the workpiece during the time interval Δt.

### 4.3. Calculating the Thickness Difference on Both Sides of the Workpiece

Assuming the width of the workpiece remains unchanged during the rolling process and only the speed changes on both sides of the steel plate are considered, *H*_1_ and *H*_2_ are denoted as the edge thicknesses on both sides of the workpiece at the inlet, while *h*_1_ and *h*_2_ are denoted as the edge thicknesses on both sides of the workpiece at the outlet. Based on the metal mass flow conservation principle at the inlet and outlet, then the following holds:

(16) $$\left\{\begin{array}{l} H_{1}V_{1}=h_{1}v_{1}\\ H_{2}V_{2}=h_{2}v_{2} \end{array}\right.$$

Assuming that $v_{2}>v_{1}$, to equalize $v_{2}$ to $v_{1}$, it is necessary to adjust the roll tilt to vary the thicknesses on both sides. By adjusting $h_{1}$ to $h_{1}^{\prime }$ and $h_{2}$ to $h_{2}^{\prime }$, the following can be derived:

(17) $$\left\{\begin{array}{l} h_{1}v_{1}=h_{1}^{\prime }\cdot (v_{1}+\frac{\Delta v}{2})\\ h_{2}v_{2}=h_{2}^{\prime }\cdot (v_{2}-\frac{\Delta v}{2}) \end{array}\right.$$

Let: $v_{1}+\frac{\Delta v}{2}=v_{2}-\frac{\Delta v}{2}$, $\Delta v=v_{2}-v_{1}$, then:

(18) $$\left\{\begin{array}{l} h_{1}^{\prime }=\frac{2h_{1}v_{1}}{v_{1}+v_{2}}\\ h_{2}^{\prime }=\frac{2h_{2}v_{2}}{v_{1}+v_{2}} \end{array}\right.$$

Then the thickness difference in the workpiece is calculated as:

(19) $$h_{2}^{\prime }-h_{1}^{\prime }=\frac{h_{2}v_{2}-h_{1}v_{1}}{\frac{v_{1}+v_{2}}{2}}=\frac{h_{2}v_{2}-h_{1}v_{1}}{\bar{v}}$$

where Δv is calculated according to Equation (15) based on the detection of the lateral displacement variation in the workpiece. Assume that the average velocity of the workpiece is $\bar{v}$, then the speeds on both sides of the workpiece at the outlet can be expressed as:

(20) $$\left\{\begin{array}{l} v_{2}=\bar{v}+\frac{\Delta v}{2}\\ v_{1}=\bar{v}-\frac{\Delta v}{2} \end{array}\right.$$

Then Equation (19) for calculating the thickness difference in the workpiece becomes:

(21) $$h_{2}^{\prime }-h_{1}^{\prime }=\frac{h_{2}(\bar{v}+\frac{\Delta v}{2})-h_{1}(\bar{v}-\frac{\Delta v}{2})}{\bar{v}}$$

**Figure 20:**
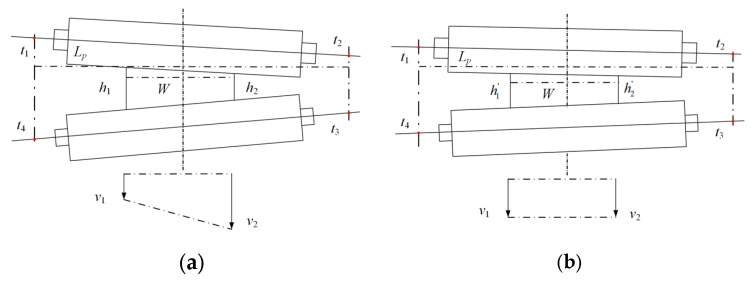
Schematic diagram before and after adjusting the roll gap of the rolling mill: (**a**) before adjustment; (**b**) after adjustment.

Sensors for rolling force, roll gap, and stiffness are located on both sides of the work rolls, with their installation positions labeled as *t*_1_, *t*_2_, *t*_3_, and *t*_4_, respectively. Among these positions, *t*_1_ and *t*_4_ are defined as the left-side (operator side) locations, while *t*_2_ and *t*_3_ are defined as the right-side (drive side) locations, and their specific arrangement is illustrated in Figure 20. Assume the distance between the installation positions of the sensors on both sides is *L_p_*, and the plate width is *W*. The left (operating side) thickness calculated from the data is *h*_os_, and the right (drive side) thickness is *h*_ds_. Based on the positions of the sensors on both sides and the width of the workpiece, it can be known that:

(22) h1=(Lp−W)2Lphds+hosh2=W+(Lp−W)2Lphds+hos

The thickness difference adjustment value of the workpiece is $\left(h_{2}^{\prime }-h_{1}^{\prime })-(h_{2}-h_{1}\right)$. Based on the proportional conversion relationship, the workpiece thickness difference can be converted into the roll gap adjustment amount, which is further extended to the adjustment amounts of the two support point positions to correct the camber.

### 4.4. Camber Straightening Control Method

**Figure 21:**
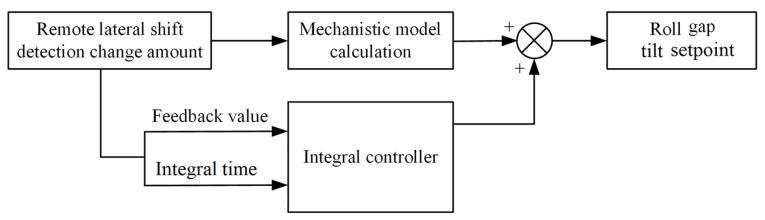
Schematic diagram of camber correction control.

To effectively control and correct the camber of heavy plates, the method of adjusting the roll gaps on both sides of the work rolls using the hydraulic cylinders of the rolling mill is usually adopted. Based on the above formula derivation, when an offset of magnitude $L_{t}$ is detected at the far end within Δ*t* time, the camber correction is achieved by adjusting the roll gap tilt amounts on both sides of the rolling mill through hydraulic oil columns, respectively. Specifically, the hydraulic cylinder on the bent side of the steel plate reduces the roll gap, while the hydraulic cylinder on the other side lifts the roll gap. This can theoretically ensure the flatness of the steel plate in subsequent rolling, thereby realizing camber adjustment. Considering the condition simplification and calculation errors in the formula derivation process, the cumulative deviation is eliminated using an integral controller [51] in actual production, as shown in Figure 21.

By detecting the far-end lateral displacement $L_{t}$, the roll gap tilt setpoint for camber control is calculated. The detection deviation is then sent to the integral controller to assist in camber correction and adjustment. The setpoint of the integral controller is that no new far-end lateral displacement occurs within Δ*t* time. The speed of deviation adjustment is ensured by setting the integral time, which dynamically eliminates the cumulative deviation in camber control during production and maintains the flatness of the steel plate during subsequent rolling.

The plate camber control is divided into two stages. Before the plate is bitten into the rolling mill, a pre-controlled roll gap tilt setpoint trained based on operational experience is adopted. After the plate is bitten into, if the far-end lateral displacement occurring during the rolling process is less than the set threshold, the feedback adjustment control model is not triggered, i.e., the original roll gap tilt setpoint is maintained consistently. When the image-based camber detection system detects that the far-end lateral displacement exceeds the threshold, the roll gap tilt setpoint is immediately updated on the basis of the original setpoint and the camber correction amount calculated by the feedback model, until the far-end lateral displacement of the steel plate is limited within the set threshold.

**Figure 22:**
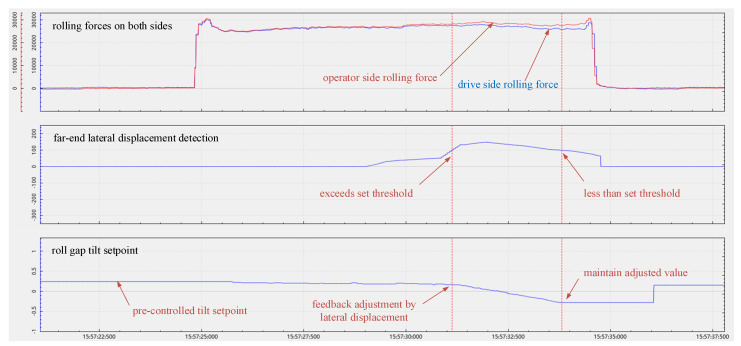
Camber control schematic diagram during the rolling process.

In the actual production process, the camber control algorithm was engineered and tested. The software adopted for the development of the camber control system is Qt platform, which fully leverages the excellent computational capability of C++ to enhance the system response. The image processing algorithm utilizes the OpenCV open-source library to achieve real-time detection of plate camber. Figure 22 presents the camber control data of the test plate, with a target thickness of 18 mm and a control threshold of 100 mm set for the far-end lateral displacement of the plate. Prior to the plate being bitten into the rolling mill, the pre-controlled roll gap tilt was derived from the output of a model trained on operational experience, which was utilized to pre-emptively eliminate camber under conventional operating conditions. As the rolling process progresses, variations in the clearance of the roll system may arise, leading to the pre-controlled roll gap tilt potentially being unable to fully suppress camber occurrence. After the plate is bitten into the mill, the far-end lateral displacement is measured using an image processing algorithm to conduct real-time evaluation of camber occurrence value. As illustrated in Figure 21, when the far-end lateral displacement of the plate exceeds the set threshold, feedback control is activated for adjustment, and an additional roll gap tilt adjustment value is calculated by the model. Once the far-end lateral displacement of the plate meets the condition of being less than the set threshold, the integral controller maintains the adjusted roll gap tilt until the completion of rolling.

Tests of camber control implemented during the rolling process demonstrate that when camber exceeds the threshold, the model can intervene and adjust in a timely manner with a closed-loop control time of less than 50 ms. By regulating the roll gap tilt, the model achieves excellent camber control performance over the entire length of the plate, effectively confining the far-end lateral displacement within the set threshold and fulfilling the expected camber control requirements.

## 5. Conclusions and Prospect

### 5.1. Conclusions

This paper takes the plate camber as the research object, conducts real-time acquisition of plate images through machine vision technology, and develops an image enhancement processing algorithm to improve the stability of camber detection in complex production environments, thereby obtaining the contour edge and centerline position information of the plate. Through data collection and model development for camber control during the rolling process, a pre-control model and a feedback control model for the plate camber have been established. Based on the integrated closed-loop control framework of perception-decision-execution, this paper addresses the complete technical chain from accurate camber measurement to real-time control, providing a validated industrial control solution for plate camber.

(1) Develop an image enhancement algorithm suitable for the complex production environment of medium and heavy plates, which significantly improves the robustness of the image detection algorithm. Aiming at the instability caused by brightness fluctuations in plate images, an adaptive grayscale algorithm is adopted to effectively enhance the quality of plate images and clearly present contour edges. For image interference caused by residual water occlusion, the iterative weighted least squares method based on Gaussian kernel function is used to accurately eliminate abnormal coordinate points and fit the center curve of plates representing the degree of camber with high precision. The application of the image enhancement algorithm significantly improves the reliability of detection data and provides a guarantee for the improvement of camber measurement accuracy.
(2) A pre-control model for plate camber based on Optuna-machine learning hyperparameter optimization has been established. This model incorporates the influence of roll system information on camber, removes abnormal data through data preprocessing and mean shift, and uses the Optuna framework to optimize the hyperparameters of SVR, DT, RF and XGBoost models. Through a comprehensive comparison of the prediction results, performance indicators, and error distribution, the XGBoost model achieves an *R*^2^ of 0.9794 and an MSE of 0.00204 on the test set, thus exhibiting the most excellent fitting performance.
(3) Using machine vision technology, a feedback control model for plate camber has been developed. Based on the automatic tracking of the far end of the plate during the rolling process, the measured value of lateral displacement at the far end of the plate is preferentially adopted as the evaluation of the degree of camber occurrence. The far end of the plate amplifies the camber measurement value, greatly improving the measurement and control accuracy. Field test results show that the control speed and accuracy of camber can meet the requirements of engineering applications. The development of the algorithm based on pre-control and camber feedback control has practical significance for guiding the online control of camber.

### 5.2. Prospect

When the dataset for training the pre-control model is insufficient in sample size or imbalanced in distribution, the camber pre-control adjustment values output by the model struggle to cover all steel grades, which increases the difficulty of feedback control. Therefore, expanding the sample size of the dataset is a crucial guarantee for improving the accuracy of the pre-control model. Meanwhile, the occurrence of plate camber is closely related to the clearance of the mill roll system. Even significant differences in roll system clearance may exist during forward and reverse rolling processes. The roll gap directly affects the exit thickness on both sides of the plate, leading to differences in elongation rates between the two sides. Feedback control technology based on machine vision is the final means to restrict the occurrence of camber, and improving the accuracy of the pre-control model can ensure that camber does not occur or occurs minimally. In the future, it is necessary to continuously collect more data during the rolling process. Based on the integration of data-driven and mechanism-based models, the ability to quickly and accurately identify equipment status will be developed. The self-organization and self-optimization capabilities of intelligent models will be utilized to find optimal camber pre-control settings, thereby further improving the control accuracy of camber.

## Figures and Tables

**Table 1:** Input and output characteristics of the data.

No.	Symbol	Definition	Unit
1	Oil_D	zero position difference	mm
2	I_T	inlet thickness	mm
3	O_T	outlet thickness	mm
4	L	workpiece length	mm
5	W	workpiece width	mm
6	V	rolling speed	m/s
7	F	rolling force	kN
8	T	workpiece temperature	°C
9	U_W_roll	upper work roll No.	-
10	L_W_roll	lower work roll No.	-
11	Camber	camber value	mm
12	Oil_S	tilt adjustment (output)	mm

**Table 2:** Environmental settings.

Software Environment	Software Versions
Anaconda	23.3.1
Jupyter	1.0.0
Python	3.9
Optuna	4.1.0
Optuna-dashboard	0.17.0
Scikit-learn	1.4.2
Xgboost	2.0.3

**Table 3:** Hyperparameter search range and optimal results.

Model	Hyperparameter	Range	Optimal Values
SVR	C	[0.1, 10]	8.66523
	gamma	[0.001, 1]	0.99851
	epsilon	[0.01, 1]	0.06468
	kernel	[‘poly’, ‘rbf’]	‘rbf’
DT	max_depth	[3, 16]	8
	min_samples_split	[2, 10]	6
	min_samples_leaf	[1, 10]	2
	max_leaf_nodes	[2, 10]	10
RF	n_estimators	[50, 500]	410
	max_depth	[3, 16]	15
	min_samples_split	[2, 10]	2
	min_samples_leaf	[1, 10]	1
XGBoost	n_estimators	[50, 500]	496
	max_depth	[3, 16]	11
	learning_rate	[0.01, 0.3]	0.16345
	subsample	[0.5, 1]	0.93806
	colsample_bytree	[0.5, 1]	0.86954
	min_child_weight	[1, 10]	1

**Table 4:** Performance evaluation comparison.

Datasets	Model	*R* ^2^	RMSE	MSE	MAE
training	SVR	0.8789	0.1086	0.01179	0.07643
	DT	0.9170	0.08989	0.00808	0.07011
	RF	0.9960	0.01977	0.000391	0.01356
	XGBoost	0.9999	0.00092	8.503 × 10^−7^	0.000645
test	SVR	0.8538	0.1203	0.01447	0.08536
	DT	0.9179	0.09016	0.00813	0.07011
	RF	0.9776	0.04771	0.00222	0.02986
	XGBoost	0.9794	0.04519	0.00204	0.02891

## Data Availability

The original contributions presented in this study are included in the article. Further inquiries can be directed to the corresponding authors.
